# Laser Tailored Multilayer Graphene Grids for Transparent Conductive Electrodes

**DOI:** 10.1186/s11671-019-3040-9

**Published:** 2019-06-18

**Authors:** Yining Jiang, Liang Gao, Xiaohan Wang, Wentao Dai, Jiang Wu, Xiao Dai, Guifu Zou

**Affiliations:** 1 College of Energy, Soochow Institute for Energy and Materials Innovations, and Key Laboratory of Advanced Carbon Materials and Wearable Energy Technologies of Jiangsu Province, Soochow University, Suzhou, 215000 China; 2 University of Electronic Science and Technology of China, Chengdu, China

**Keywords:** Multilayer graphene, Laser tailoring, Graphene grid, Efficient defogger

## Abstract

Applications of graphene as transparent conductive electrodes (TCE) have been hindered either by high cost of single crystal graphene or balance between transparency and sheet resistance of polycrystalline graphene. In this work, we propose to fabricate multilayer graphene film grids (MGFG) to enhance transparency and keep low sheet resistance through IR laser tailoring. It is proved that the transparency of MGFG could be increased by 200 times while remaining its competitive sheet resistance as low as 340 Ω sq^−1^ through adjusting the tailoring grid, and the corresponding figures of merit (FoM) is increased from 0.1 to 3.6. As-obtained MGFG is demonstrated in generating controllable local thermal field and defogging efficiently. The strategy of laser-tailoring grid will greatly advance the applications of graphene for transparent electrodes in industry.

**Electronic supplementary material:**

The online version of this article (10.1186/s11671-019-3040-9) contains supplementary material, which is available to authorized users.

## Introduction

Graphene has been highly prized as candidate for TCE for its outstanding electric and optic properties [1–6]. Large scale and single crystal graphene deposited on metal substrate through chemical vapor deposition (CVD) method shows excellent transparency (~ 97%) and conductivity (< 100 Ω sq^−1^) [7, 8]. However, the relative low growth speed and transfer process increase the massive production cost and hinder industrial application. In order to decrease the massive production cost, great works have been done for depositing polycrystalline graphene directly on commercial glass and attempted to apply for electric thermal devices, cell culture, smart window, and touch panel [9–13]. Although the growth speed has been greatly advanced, the conductivity of the polycrystalline graphene decreases a lot than the single crystal graphene. On the one hand, the graphene film with ~ 95% transmittance shows sheet resistance up to 6.1 kΩ sq^−1^, on the other hand, the transmittance will be decreased below 50% due to thickness increment upon a sheet resistance below 0.5 kΩ sq^−1^ [14–17]. Therefore, there is still a big challenge to balance the competition between sheet resistance and transmittance for the graphene film. Herein, we proposed a laser-tailoring route for fabricating graphene grids to realize the high transparency and good conductivity of multilayer graphene film (MGF). IR laser is applied to partially ablate the multilayer graphene and tailor the thin film to desirable pattern. The film transparency is remarkably increased from 0.38 to 75% while maintaining the sheet resistance as low as 350 Ω sq^−1^ through adjusting aperture size or gird width. It is worth noting that the laser tailoring process is rather rapid that tailoring of 5 cm × 5 cm thin film can be finished within 1 min, which guaranteeing wide application for large scale in industry. We demonstrate efficient defogger based on MGFG as well as controllable local thermal field on substrate through designing of the grids’ patterns. The highly transparent and conductive MGFG will have great potential applications as transparent electrodes in touch panel, smart window, and wearable devices.

## Results and Discussion

**Fig. 1:**
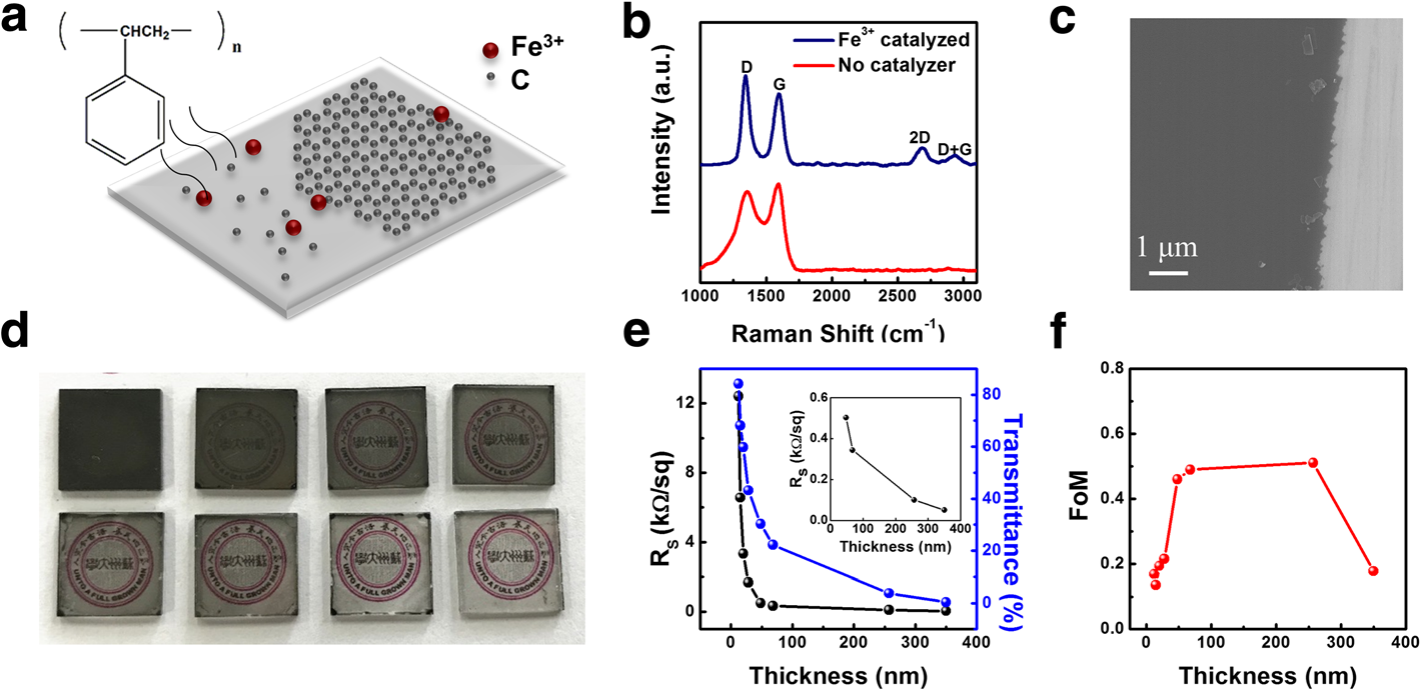
Deposition and characterization of MGF. **a** Schematic illustration of CVD deposition of MGF with Fe^3+^ as catalyzer. **b** Raman spectrum of graphene film with and without catalyzer (at 633 nm excitation wavelength). **c** SEM image of MGF. **d** Photos of MGF deposited on quartz substrate with different thickness. **e** Comparison of the sheet resistance and transmittance of MGF with different thickness. **f** Comparison of the thickness and FoM of MGF obtained in this work

Initially, MGF with different thickness are deposited on transparent quartz substrate through chemical vapor deposition method. Herein, polystyrene (PS) is applied as carbon source that is evaporated at 300 °C and deposited onto substrate at 1000 °C under Ar/H_2_ atmosphere. In order to assist the growth of multilayer graphene, Fe ions that coordinated with polyethyleneimine are spun and coated on substrate serving as catalyzer (Fig. 1a)**.** During the annealing process, Fe ions aggregate each other and transform into Fe nanoparticles in the film. Additional file 1: Figure S1 presents the different Fe^3+^ concentration influencing on morphology and crystallization of MGF (Additional file 1: Figure S1, Supporting information). To secure the quality of MGF, 0.5 mg/ml Fe^3+^ is optimum to grow high-density graphene films. It is found that the Raman spectrum of the deposited film without Fe catalyzer (Fig. 1b) does not contain the representative 2D and D+G bands of graphene but broad G and D bands. Nevertheless, with the assistance of the Fe catalyzer on substrate, the corresponding Raman spectrum shows obvious 2D band at 2684 nm and D+G band at 2933 nm except for D band at 1342 nm, G band at 1592 nm, which indicates the deposited thin film is characteristics of graphene [18, 19]. The scanning electron microscope (SEM) image in Fig. 1c clearly exhibits high density and smoothness of MGF. MGF with different thickness are fabricated through adjusting quantity of the PS quantity (Fig. 1d, e). It could be seen that both film sheet resistance and transmittance sharply drop with increasing film thickness. Three-nanometer-thick thin film has high transparency with 80% transmittance at 550 nm but poor conductivity of a sheet resistance of 13.5 kΩ sq^−1^, while the film resistance of 0.1 kΩ sq^−1^ corresponds to an astonishingly low transmittance of 0.38%. Usually, the quality factor FoM is introduced to evaluate relativity between resistivity and transparency of the MGF as transparent electrodes. FoM is calculated via Eq. (1) where transmittance and sheet resistance are *T* and *R*_*s*_, respectively.

1 $$ \mathrm{FoM}=\frac{188.5}{Rs\left(\sqrt{\frac{1}{T}}-1\right)} $$

Hererin, FoM of the MGFs with different thickness from 10 nm to 350 nm could be calculated from 0.1 to 0.5 in Fig. 1f, which is comparable to the reported exfoliated graphite [11, 16].

**Fig. 2:**
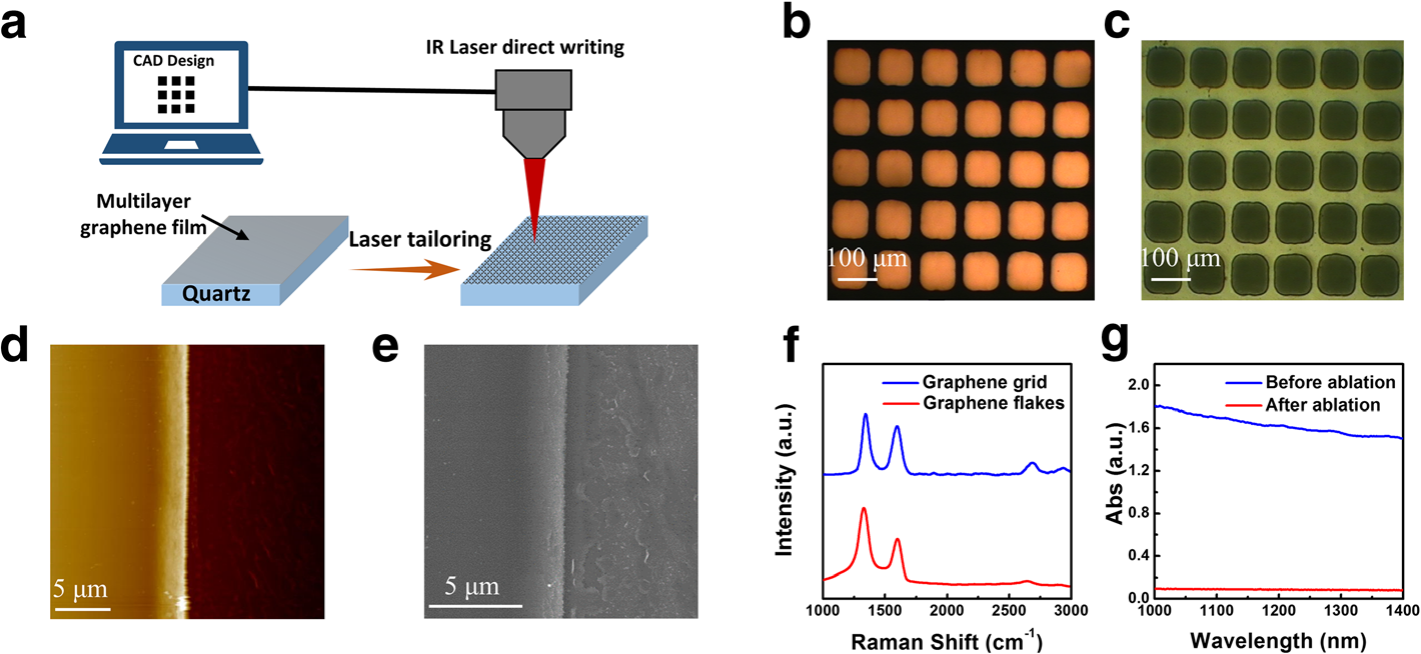
Laser tailoring of MGF and fabrication of MGFG. **a** Schematic illustration of graphene grid ablation process by IR laser direct writing. **b**, **c** Microscope images of the fabricated MGFG at transmission mode and reflection mode, respectively. **d**, **e** AFM and SEM images of tailored edge. **f** Raman spectrum of graphene grid and flakes in the ablation area ( at 633 nm excitation wavelength). **g** IR absorption of MGF before and after ablation

How to improve the FoM of as-grown MGF? The most important thing is to balance the contradiction between transparency and sheet resistance described above. Herein, IR laser was applied to ablate MGF for creating micro-grids structures (Fig. 2a). The tailoring process is based on the mechanism that the film absorbs the powerful energy from the highly focused laser beam and transforms highly dense thermal energy resulting in ablated instantly on the beam radiation site [20, 21]. With the assistant of a laser direct writing system, the multilayer graphene thin film could be tailored into arbitrary patterns (Additional file 1: Figure S2) by finely tuning laser power, scanning speed, and beam diameter. The feature width of the tailoring trace is optimized from 25 μm to 100 μm, and the minimum pattern width is up to 5 μm. To obtain optimum FoM, the grid structure of screen window is fabricated in Fig. 2b, c. It can be seen that well-organized microstructures are presented in microscopic images of the fabricated MGFG at transmission mode and reflection mode respectively. The tailored micropores are uniform and transparent, meanwhile the remaining grids are connective. SEM images in Additional file 1: Figure S3 illustrate the details of graphene films structure including micropores and grids. The micropore size is around 100 μm. Figure 2d, e shows the straight and sharp edge of MGFG in the AFM and SEM images. It proves the tailoring process is very effective to manufacture high-quality patterns. Figure 2f shows the Raman spectrums of the tailored grids that the remaining grids maintain the original structure of MGFG without deterioration after the tailoring process, while the residual flakes shows relative higher D band and weaker 2D band due to the laser ablation process [18]. Further study of infrared absorption is carried out before and after ablation of MGFG. There is no obvious absorption for ablated MGFG in Fig. 2g, which suggests that the graphene layers can be well removed by the laser ablation.

**Fig. 3:**
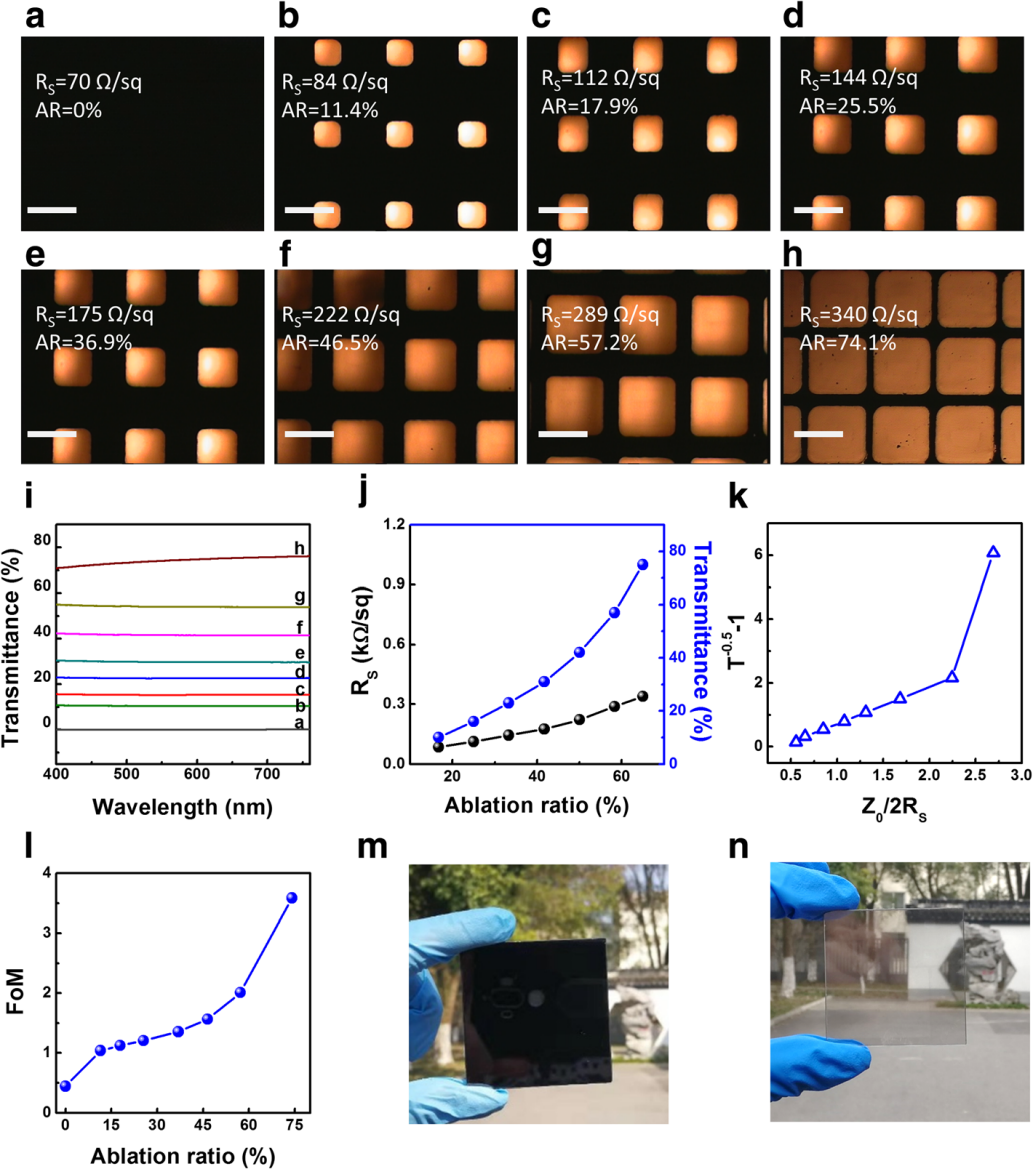
Characterization of MGFG with different ablation ratio. **a**–**h** Microscope images of MGFG with different ablation ratio. Scale bars 200 μm. **i** Transmittance of MGFG with different ablation ratio. **j** Comparison of the sheet resistance and transmittance of MGFG with different ablation ratio. **k** *T* and *R*_*S*_ data for MGFG with different ablation ratio. **l** FoM of MGFG with different ablation ratio. **m**, **n** Photographs of 5 cm × 5 cm graphene film sample before and after laser tailoring

To evaluate the influences on transmittance and sheet resistance from tailored grids parameters, we carried out a series of MGFG with different ablation ratio from Fig. 3a–h. The micropore size is finely adjusted from 100 μm × 100 μm to 250 μm × 250 μm, and the line width is tuned from 180 μm to 30 μm. As the ablation ratio increases from 0 to 75%, the transmittance increases from 0.38 to 75% and the sheet resistance increases from 70 Ω sq^−1^ to 340 Ω sq^−1^ in Fig. 3i–j. Additionally, different resistivity, micropore size, and grid width of MGFs (Additional file 1: Figure S4) are well conducted to study the optimum results between transparency and sheet resistance. In Fig. 3k–l, it could be estimated that the transmittance has been increased as much as 200 times while the sheet resistance increases only 5 times, and the FoM is increased from 0.4 to 3.6. Comparing grids with the MGF at a transmittance of 80%, the FoM is around 0.1 in Fig. 1e. Meanwhile, the sheet resistance of the graphene grids is 340 Ω sq^−1^, which is only 2.5% of MGF (13.5 kΩ sq^−1^). That is to say, the FoM of the MGFG is increased as high as 3.6 from 0.1 of MGF under the equal transmittance of 80%. Therefore, it could be firmly concluded that the transparency and conductivity of MGFG have been dramatically enhanced than MGF through tailoring into micro-grid. To demonstrate the visual effect, a 5 cm × 5 cm MGF sample is presented in natural light. The sample in Fig. 3m is totally opaque. It is worth noting that the transparency of the sample is dramatically improved after laser tailoring. The clear landscape appears through the sample of MGFG in Fig. 3n.

**Fig. 4:**
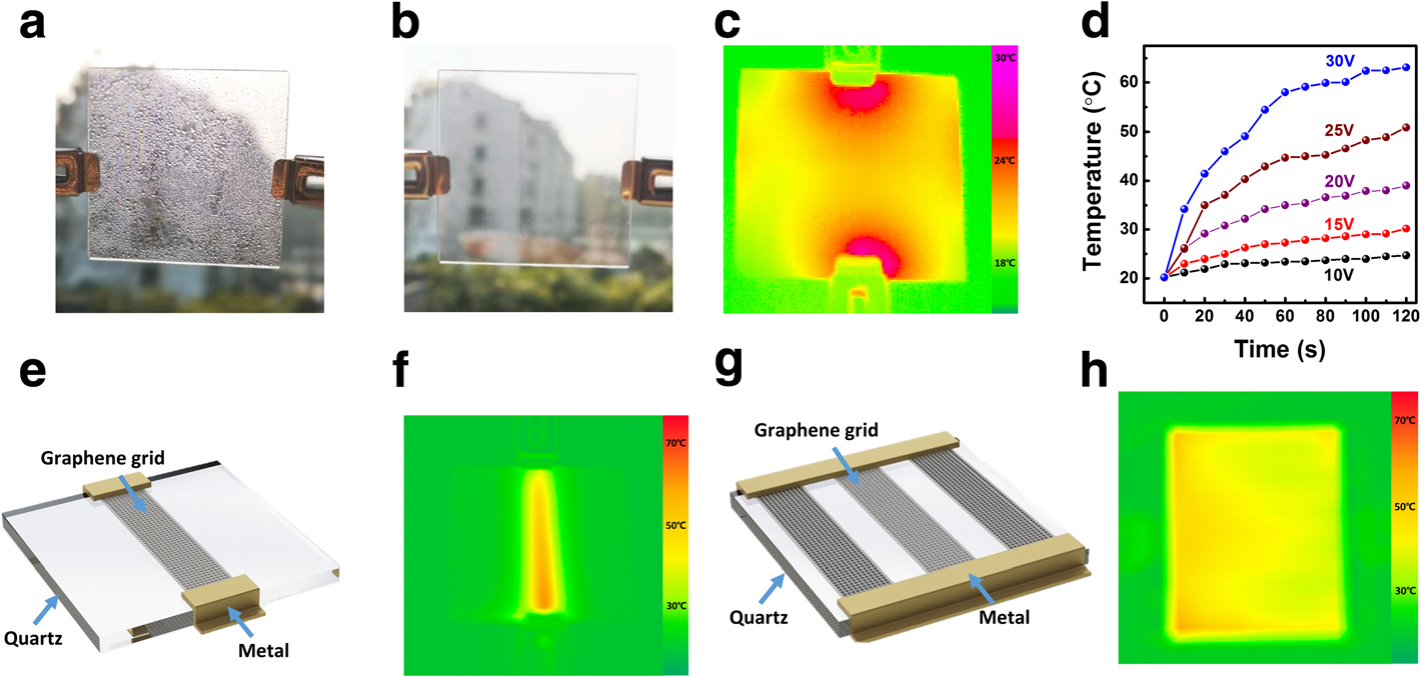
Defogger based on MGFG. **a**, **b** Defogging performance of MGFG. **c** Contour temperature map regarding the surface of 5 cm × 5 cm MGFG under 20 V. **d** Temperature profiles of 1 cm × 1 cm MGFG at different voltage and time. **e** Schematic illustration of MGFG belt defogger. **f** Contour temperature map of MGFG belt defogger under 25 V. **g** Schematic illustration of patterned MGFG belt arrays defogger. **h** Contour temperature map of MGFG belt arrays defogger under 25 V

For demonstrating applications of the MGFG, Fig. 4a, b shows that as-fabricated grids on a quartz substrate are utilized as transparent electrical-thermal defogger. The electrical-thermal performance of the grids with 75% transmittance is studied at different voltages. It is interesting to see that much water drops on the surface of grids (Fig. 4a) are gone within 2 min when power is on in Fig. 4b. To identify the process, contour temperature map of MGFG in Fig. 4c is used to directly investigate the electrical-thermal behavior. Figure 4d shows that the surface temperature of MGFG increases with increasing time and voltage. It is found that voltage much influences on the temperature of MGFG. At the same voltage, the temperature sharply increases at the first stage and then tend to be stable. Further investigation finds that there is more thermal aggregation around two-point electrodes in Fig. 4c. The accumulated thermal field mainly arises from the inhomogeneous distribution of electrical current density. The two contacting electrodes have a higher current density than other place of a defogger, which induces higher temperature. Base on this mechanism, the current density of the defogger could be homogenously distributed to realize the localized and controllable thermal field on the substrate through tailoring MGFG into desirable patterns. We designed a belt of MGFG through tailoring graphene grids on substrate as illustrated in Fig. 4e. The resultant contour temperature map of MGFG belt exhibits a localized thermal filed on substrate (Fig. 4g). Subsequently, one array of MGFG belt is ideally designed to homogenously conduct electricity in Fig. 4h. The experiment demonstrates a uniform thermal field on substrate can be obtained in Fig. 4h through utilizing planar electrodes and grids belt arrays on substrate. It is greatly helpful to fabricate the electrical-thermal device with high quality in the coming future.

## Conclusion

IR laser is utilized to transform non-transparent MGF into highly transparent and conductive electrodes through tailoring micro-grids structures. Arbitrary multilayer graphene patterns could be obtained under the help of the CAD design and laser direct writing system. It is worth noting that the tailoring process is rather fast for large-scale manufacturing desirable structure. The transparency of the well-maintaining conductive MGF could be significantly enhanced from 0 to 80% through partially ablation and creation of micro-grids. Applications of the MGFG are demonstrated for electrical-thermal device and controllably localized thermal field on the substrate through designing the grid patterns. This route of fabricating graphene grids is effective to open up the possibility for the multilayer graphene or even graphite film for directly being utilized as transparent electrodes without complicated exfoliation process.

## Methods

The precursor of aqueous Fe^3+^ ion catalyzer is prepared by adding 2.5 g Fecl_3_ to a solution containing 1 g polyethyleneimine (PEI), 1 g ethylenediaminetetraacetic acid (EDTA), and 30 mL water. After ultrafiltration, the final Fe concentration was 28.20 mg/mL measured by an inductively coupled plasma atomic emission spectrometer (ICP-AES, PerkinElmer Optima 8000). The solution with a concentration of 28.20 mg/ml Fe^3+^ is diluted into 0.5 mg/ml and then spin-coated onto quartz substrates at 5000 rpm for 30 s. The films were annealed at 1000 °C for 10 min with Polystyrene (PS) put at one side of the tube as carbon source.

Graphene grid is tailored by 1064 nm IR laser (YDFLP-20-M1+-S) provided by JPT Electronics at scanning speed of 100 mm/s, power of 2 W, frequency of 42 Hz and pulse width of 100 ns.

### Characterizations

Raman spectra were collected from Horiba Jobin Yvon HR Evolution. Scanning electron microscopy (SEM) analysis was carried out on a FEI Scios, operating at 10 kV. The optical image was obtained from Metallographic microscope CMM-55E. Sheet resistance was tested by the four-probe tester ST2263. Transmittance was tested on a Shimadzu UV-2450. Contour temperature map was measured by an Infrared camera (VarioCAM) from InfraTec.

## Additional file

Additional file 1: Supporting information is available online. (DOC 12218 kb)

## Data Availability

All data generated or analyzed during this study are included in this article.

## Abbreviations

CVD: Chemical vapor deposition
EDTA: Ethylenediaminetetraacetic acid
FoM: Figures of merit
MGF: Multilayer graphene film
MGFG: Multilayer graphene film grids
PEI: Polyethyleneimine
PS: Polystyrene
SEM: Scanning electron microscope
TCE: Transparent conductive electrodes
