# Supplementary material for: Laser Tailored Multilayer Graphene Grids for Transparent Conductive Electrodes
Source: Nanoscale Res Lett. 2019 Jun 18;14:207. doi: 10.1186/s11671-019-3040-9 (PMC6582020; doi:10.1186/s11671-019-3040-9)
Supplement: Supplementary file 1 — Supporting information is available online. (DOC 12218 kb) [file 11671_2019_3040_MOESM1_ESM.doc]

Supporting Information

**Laser Tailored Multilayer Graphene Grids for Transparent Conductive Electrodes**

Yining Jiang†^1^, Liang Gao†^1^, Xiaohan Wang^1^, Wentao Dai^1^, Jiang Wu^2^, Xiao Dai*^1^ and Guifu Zou*^1^

Yining Jiang: 20164208125@stu.suda.edu.cn;

Liang Gao: 20174208091@stu.suda.edu.cn

Xiaohan Wang: 20174208056@stu.suda.edu.cn

Wentao Dai: 1073712628@qq.com

Jiang Wu: jiangwu@uestc.edu.cn

Xiao Dai: daixiao@suda.edu.cn

Guifu Zou: zouguifu@suda.edu.cn

**
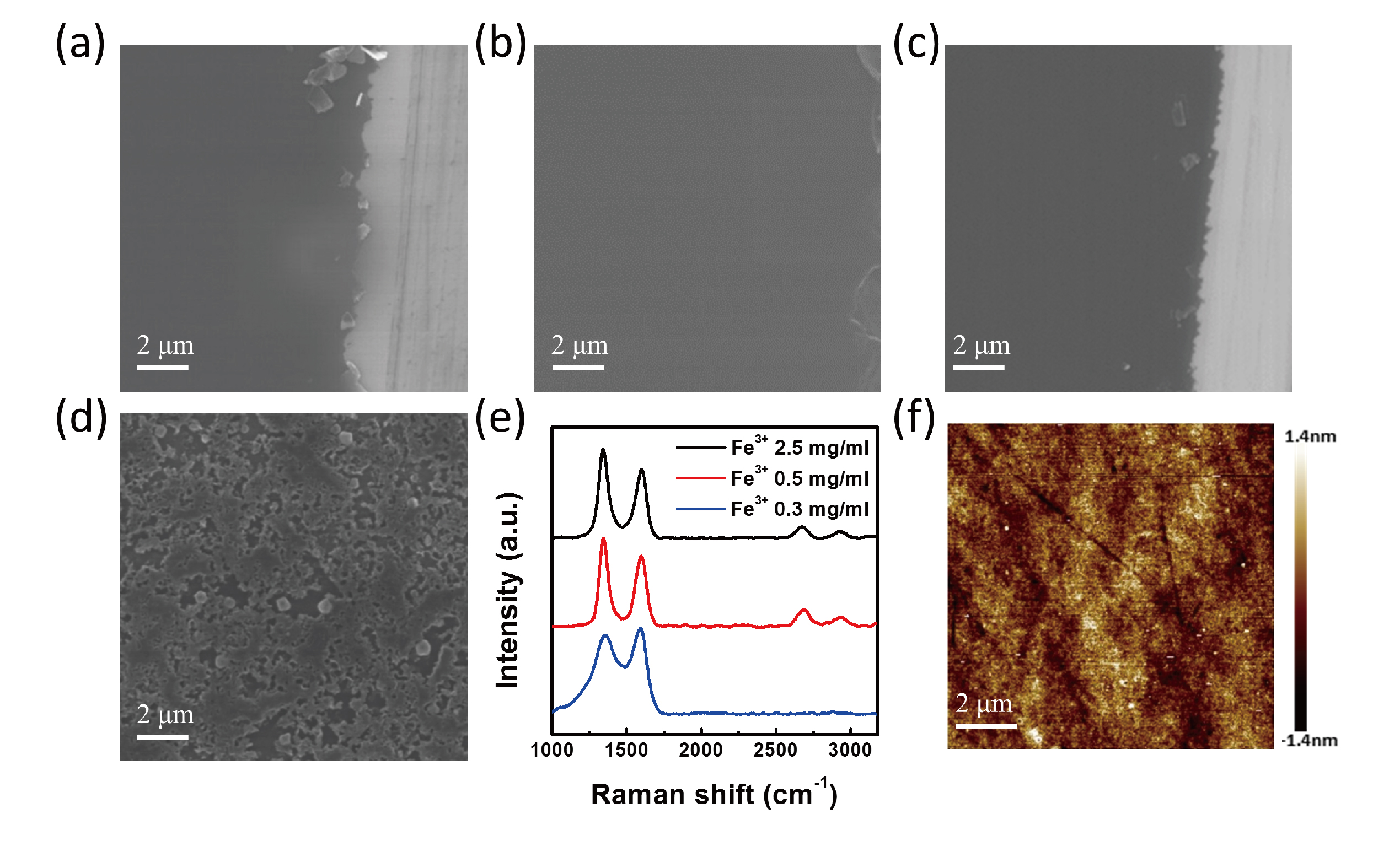
**

**Figure S1.** (a) SEM image of graphene film without catalyzer. (b) SEM image of graphene film catalyzed by 0.3 mg/ml Fe^3+^. (c) SEM image of graphene film catalyzed by 0.5 mg/ml Fe^3+^. (d) SEM image of graphene film catalyzed by 2.5 mg/ml Fe^3+^. (e) Raman spectrum of graphene catalyzed by Fe^3+^ with different concentration. (f) AFM image of graphene surface catalyzed by 0.5 mg/ml Fe^3+^.


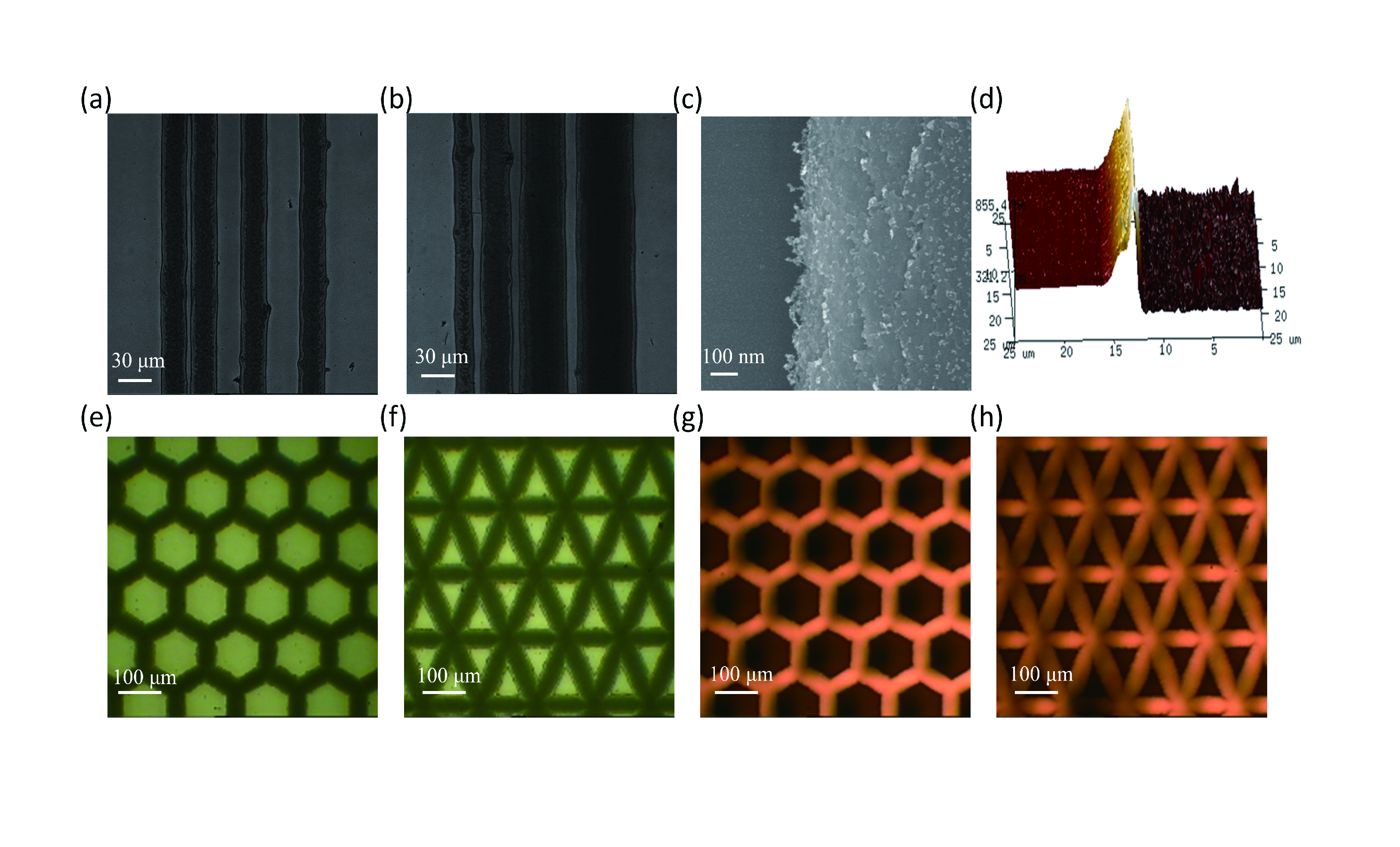


**Figure S2.** (a) Tailored line pattern with different interval. (b) Tailored line pattern with different width. (c) SEM image of graphene film grid edge. (d) AFM 3D image of graphene film grid edge. (e-f) Optical images of different tailored pattern in reflection mode. (g-h) Optical images of different tailored pattern in transmission mode.


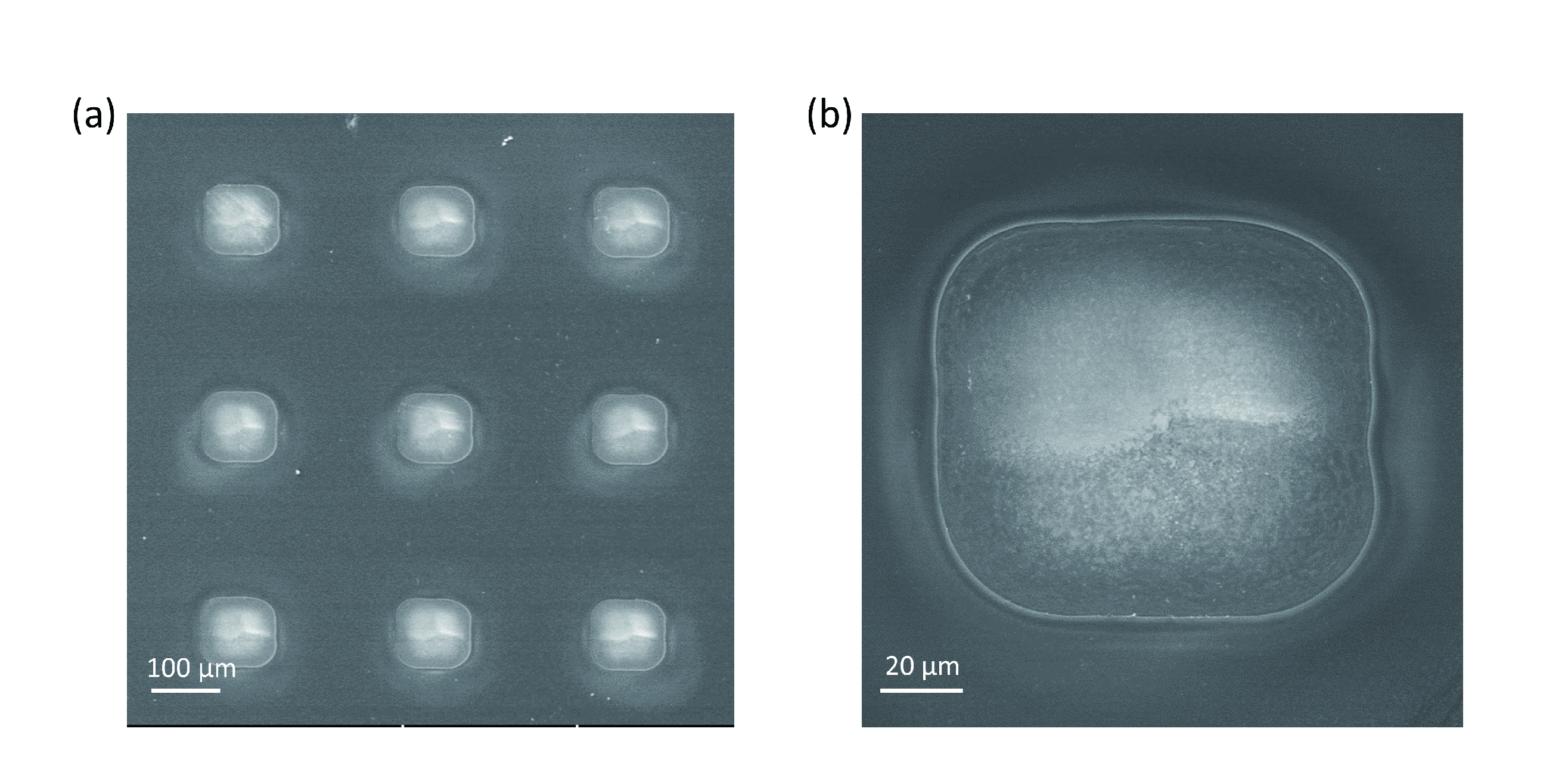


**Figure S3.** SEM images of (a)graphene grid(b) micropore structure at high-magnification.


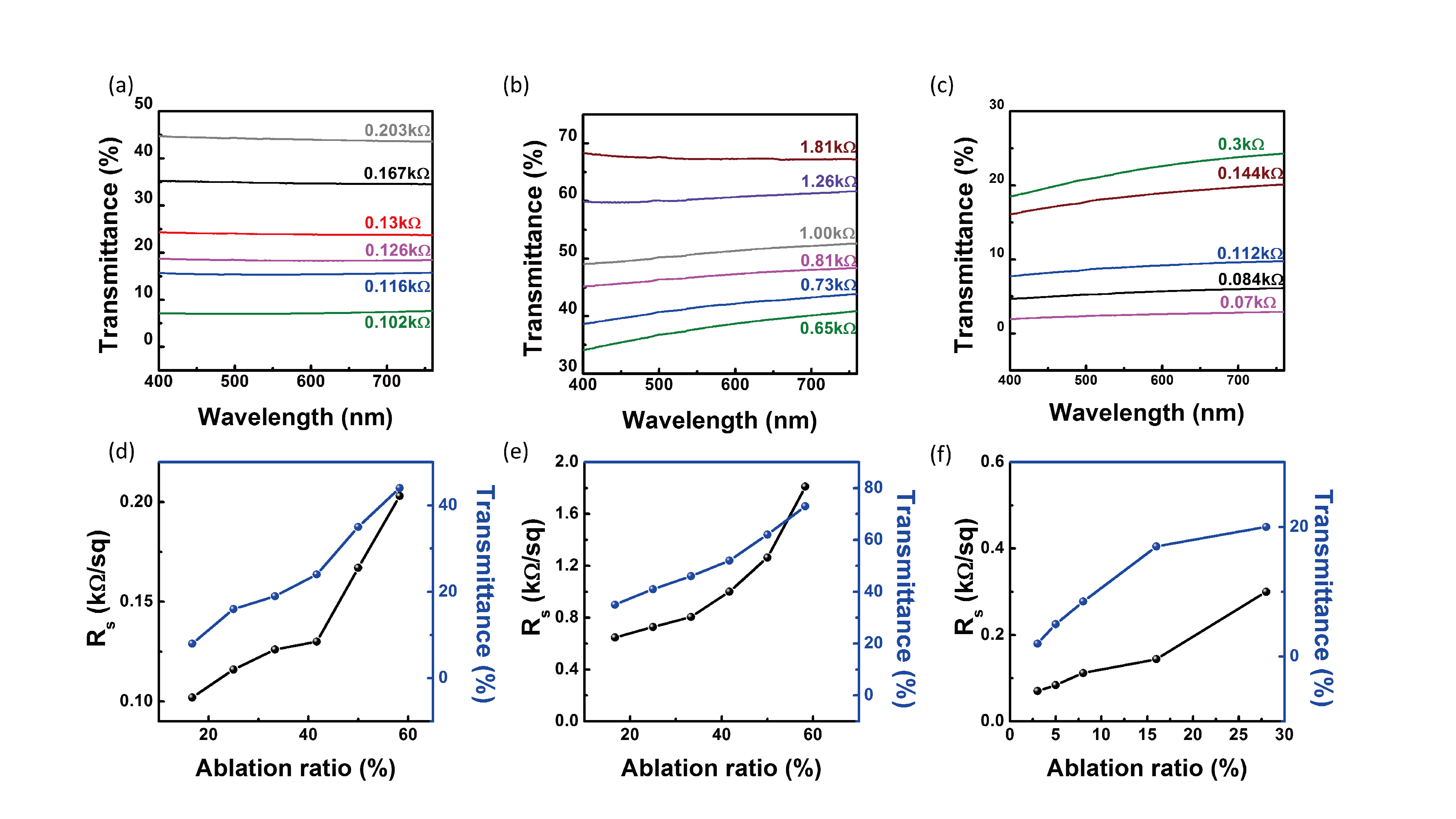


**Figure S4.** Transmittance and R_S_ of graphene grid at different ablation ration. (a, d) Initial film is 70 Ω·sq^−1^. After laser ablation, the micropore size is finely adjusted from 100 μm×100 μm to 250 μm×250 μm, and the line width is tuned from 360 μm to 60 μm. (b, e) Initial film is 500 Ω·sq^−1^, after laser ablation, the micropore size is finely adjust from 100 μm×100 μm to 250 μm×250 μm, and the line width is tuned from 180 μm to 30 μm. (c, f) Initial film is 70 Ω·sq^−1^. After laser ablation, micropore size is kept as 100 μm×100 μm and the line width is tuned from 360 μm to 100 μm.


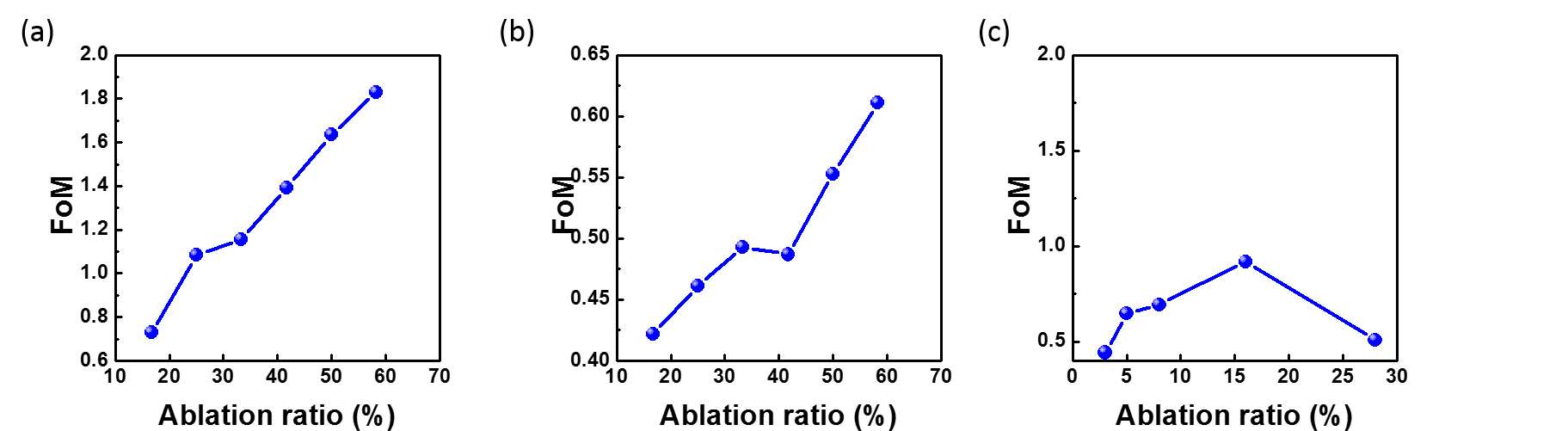


**Figure S5.** FoM obtained by different resistivity, micropore size, and grid width of MGFGs. (a)FoM of MGFG corresponding to the data from Figure S4(a,d) (b)FoM of MGFG corresponding to the data from Figure S4(b,e) (c) FoM of MGFG corresponding to the data from Figure S4(c,f).

Comparing the result showed in Figure S5 to the optimum result exhibited in Figure3, it can be concluded that FoM is similar when changing micropore size and grid width at the same time if keeping the ablation ratio unchanged. And with higher resistance of initial film, MGFG exhibits lower FoM at the same ablation ratio. Besides, changing ablation ratio by remaining the micropore size unchanged results in rather low FoM.
